# Virtual reality in neurorehabilitation for musculoskeletal disorders: a bibliometric analysis from 2010 to 2025

**DOI:** 10.3389/fneur.2026.1922187

**Published:** 2026-09-09

**Authors:** Lei Zhang, Xiaoyue Zhang, Rui Zhang, Yong Yu, Zhen Yang, Lin Yang

**Affiliations:** 1 Rehabilitation Medicine Center and Institute of Rehabilitation Medicine, West China Hospital, Sichuan University, Chengdu, China; 2 Key Laboratory of Rehabilitation Medicine in Sichuan Province, West China Hospital, Sichuan University, Chengdu, China; 3 Institute of Life Course and Medical Sciences, University of Liverpool, Liverpool, United Kingdom; 4 Department of Movement Sciences, KU Leuven, Leuven, Belgium

**Keywords:** bibliometric analysis, chronic pain, digital therapeutics, musculoskeletal disorders, rehabilitation, virtual reality

## Abstract

**Introduction:**

Virtual reality has become increasingly important in neurorehabilitation and has shown considerable potential for the management of musculoskeletal disorders. Although an increasing number of systematic reviews and meta-analyses have summarized the effectiveness of VR interventions for musculoskeletal disorders, a comprehensive overview of the development of this research field is still lacking. Therefore, this bibliometric analysis aimed to identify the knowledge structure and emerging research hotspots in the field of virtual reality for musculoskeletal disorders from 2010 to 2025.

**Methods:**

Publications were retrieved from the two databases, Web of Science Core Collection and Scopus. After merging the databases and removing duplicates, two independent reviewers screened the titles and abstracts according to predefined criteria. Finally, a total of 433 eligible publications were included for bibliometric analysis. BibliometriX and VOSviewer were used to identify knowledge structure and emerging research hotspots in this research field. A LOESS curve was applied to visualize the trend of the annual number of publications over time. The BIBLIO reporting checklist was used to enhance transparency and reproducibility.

**Results:**

The annual number of publications increased steadily from 2010 to 2025, with rapid growth after 2019, reaching a peak of 97 publications in 2025. A total of 1,878 authors from 60 countries contributed to at least one publication in this field. The USA produced the largest number of publications, followed by several European countries. Keyword analysis identified chronic pain, rehabilitation, and low back pain as major research topics. Thematic evolution indicated a shift from early technological exploration toward clinically oriented research focusing on rehabilitation outcomes, pain management, and the neurophysiological mechanisms underlying immersive VR interventions.

**Conclusion:**

This bibliometric analysis provides a comprehensive overview of the development of VR for musculoskeletal disorders and highlights an ongoing transition from technology-driven exploration to clinically oriented and mechanism-informed research. These findings may help guide future interdisciplinary research and support the development of more evidence-based VR rehabilitation strategies.

## 1 Introduction

Musculoskeletal disorders (MSDs) are among the most prevalent causes of disability worldwide and impose a substantial socioeconomic burden on global health systems (1). Moreover, the number of people living with MSDs worldwide is projected to reach approximately 2.2 billion by 2035, accompanied by a trend toward younger age at onset (1). Chronic conditions such as low back pain, and work-related musculoskeletal injuries are often associated with persistent pain, functional limitations, and long-term medical needs (2). Although physical therapy (e.g., manual therapy and sports rehabilitation) and pharmacological treatments (e.g., non-steroidal anti-inflammatory drugs) have demonstrated to be effective in relieving pain associated with MSDs, maintaining long-term functional improvement remains challenging (3, 4).

With the development of technology, virtual reality (VR) has emerged as a promising rehabilitation approach by integrating visual, auditory, and sometimes haptic stimuli to enhance patient engagement, facilitate motor learning, and modulate pain perception through attentional and cognitive mechanisms (5–7). The clinical application of VR has been well established in neurological and pediatric rehabilitation (8, 9), where commercial game-based VR systems (e.g., Nintendo WII and Xbox Kinect) have been successfully integrated into rehabilitation programmes for individuals with stroke (10), and cerebral palsy (11). These systems have been shown to improve motivation, increase therapy intensity, and achieve functional outcomes comparable to conventional rehabilitation while offering greater accessibility and patient engagement (12). Furthermore, recent rehabilitation models increasingly integrate VR with telerehabilitation and digital therapeutics, highlighting its potential to deliver personalized and remotely supervised rehabilitation across diverse clinical populations (13, 14).

Building upon these successful clinical applications, VR has been increasingly adopted for musculoskeletal rehabilitation and pain management. For example, a recent study demonstrated that non-immersive VR–based exercise significantly reduced the pain level while improving muscle excitability and balance in patients with knee osteoarthritis compared with conventional physiotherapy (15). Moreover, accumulating evidence from recent clinical studies and systematic reviews suggests that VR interventions can reduce pain, improve functional outcomes, and enhance rehabilitation adherence among individuals with MSD, supporting their role as a complementary or alternative therapeutic approach (16, 17). Furthermore, research on VR interventions for MSD has expanded steadily over the past two decades, progressing from early feasibility studies toward randomized controlled trials and remote rehabilitation models (17).

However, despite the rapid increase in publications and the expanding clinical applications of VR for MSD, research in this field remains highly heterogeneous with respect to intervention types, target conditions, and research methodologies. Existing reviews primarily focus on evaluating the clinical effectiveness of VR interventions for specific disorders, frequently employing systematic (with or without meta-analyses) or narrative review methodologies. Whilst these approaches are crucial for evaluating treatment effectiveness and informing policy formulation, they are limited in capturing the overall research trends and hotspots within the research field of VR for MSD. This includes difficulties in comprehensively presenting publication trends, collaborative networks, and the evolution of research topics (18).

Bibliometric analysis provides a quantitative approach to address these limitations by mapping the knowledge structure of a research field (18, 19). Based on citation-based and co-occurrence analyses, bibliometric analysis can detect emerging research hotspots (18). This method has been employed in the fields of VR in health care (20), and VR for pain (21). To date, however, no bibliometric analysis has examined publications in the research field of VR for MSD. Given the accelerating growth of publications in this field and its increasingly significant clinical importance, there is a need for a rigorous bibliometric analysis in the research field of VR for MSD. Therefore, the aim of this study is to identify the knowledge structure and emerging research trends in this research field from 2010 to 2025. Specifically, this study aims to (1) characterize trends in scientific productivity; (2) identify the most prolific authors, countries, and journals; and (3) identify research trends and hotspots.

## 2 Methods

### 2.1 Study design

This bibliometric analysis was conducted in strict adherence to the step-by-step bibliometric guidelines (18), utilizing the BIBLIO reporting checklist to enhance transparency and reproducibility (22). The eligibility criteria for publications were formulated according to the PECO framework (23), where the population (P) included individuals with MSD, the exposure (E) referred to VR interventions, the outcomes (O) included all health-related outcomes, and no restrictions were placed on the comparator (C).

### 2.2 Data source and search strategy

A systematic literature search was conducted in two multidisciplinary databases: the Web of Science Core Collection (WoSCC) and Scopus. These multidisciplinary databases provide broad coverage of peer-reviewed scientific literature and are commonly used in bibliometric analyses (24, 25). Four indexes from WoSCC were included in the search, namely the Science Citation Index Expanded, Social Sciences Citation Index, Arts & Humanities Citation Index, and Emerging Sources Citation Index. The search period was set from 1st January 2010 to 31st December 2025. Because research on VR for MSD involves multiple disciplines, two multidisciplinary databases were selected to provide broader coverage of the relevant literature (24, 26). The search strategy was developed through discussions among the authors and with reference to previous reviews in this research field (27, 28). The search query consisted of two groups of keywords related to VR and MSDs, which were connected using the Boolean operators “OR” within each group and “AND” between the two groups. The detailed search strategy is provided in Supplementary materials 1. The search was applied to titles and author keywords.

### 2.3 Eligibility criteria and study selection

**Figure 1:**
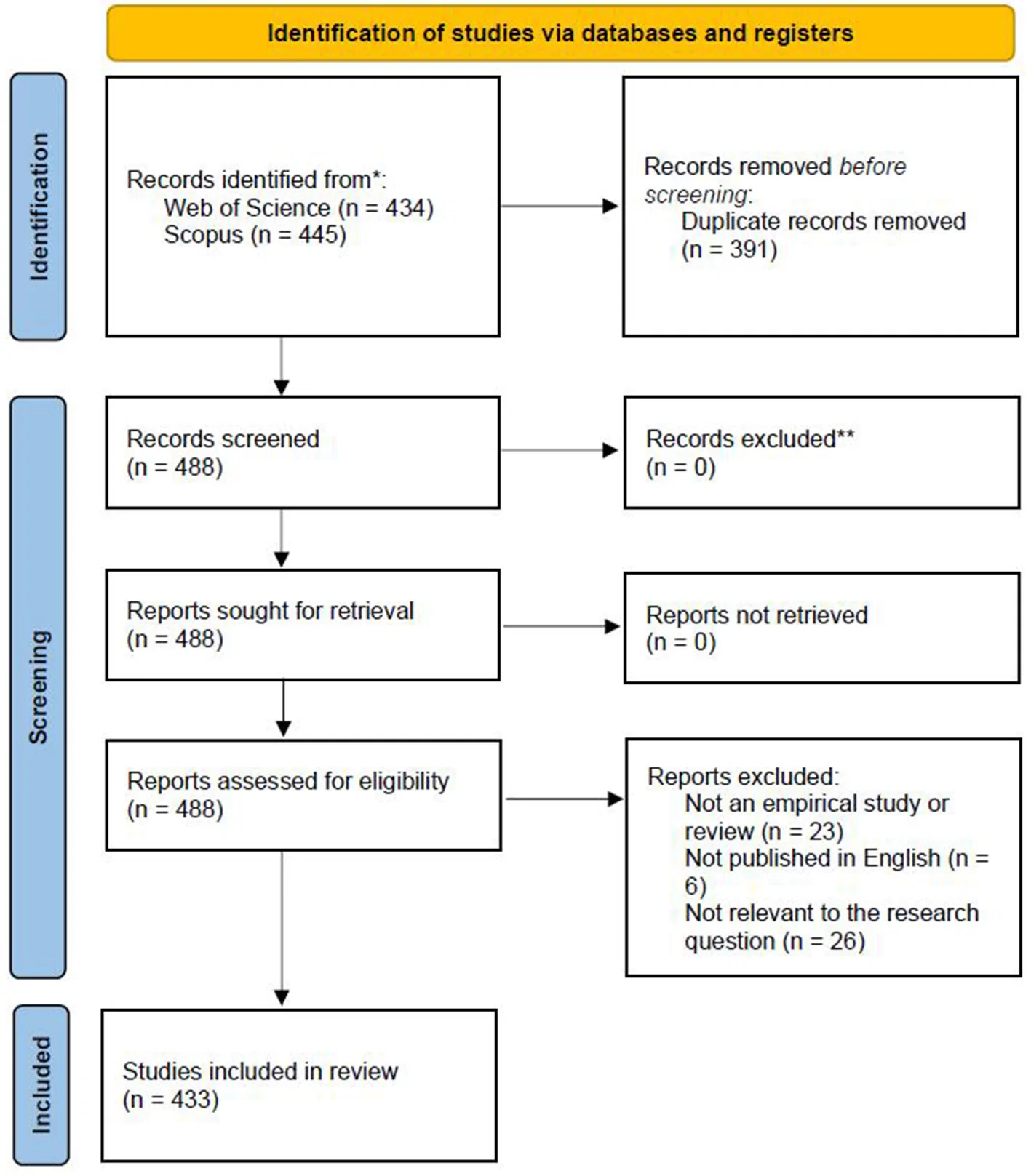
Flow diagram of the study selection process.

The eligibility criteria were defined based on the PECO framework and applied during the screening process. Studies were considered eligible if they investigated VR interventions among individuals with MSD and reported at least one health-related outcome. Only empirical studies and reviews written in English were included to ensure the consistency and comparability of bibliometric metrics. Studies were excluded if they were not related to MSD, did not involve VR interventions, were conference abstracts, editorials, letters, book chapters, or notes, or were duplicate records identified during the database merging process. All records retrieved from the WoSCC and Scopus were exported and merged using the BibliometriX package in R. A total of 878 records were initially retrieved, including 433 records from WoSCC and 445 records from Scopus. After merging the datasets and removing duplicates, 488 unique records remained. Subsequently, records that were not classified as empirical studies or reviews were excluded, leaving 465 publications. After removing non-English publications, 459 records remained for further screening. The titles and abstracts of these records were independently screened according to the predefined eligibility criteria by two individual reviewers (LZ and RZ), and 26 records that did not meet the eligibility criteria were excluded. Finally, 433 publications were included in the final bibliometric analysis, consisting of 391 records from WoSCC and 42 records from Scopus. Figure 1 presents the study selection process using a PRISMA flow diagram solely to improve the transparency of literature retrieval, duplicate removal, eligibility assessment, and record inclusion, rather than to guide the synthesis or reporting of clinical intervention findings.

### 2.4 Data extraction and bibliometric analysis

Bibliographic metrics of the included publications were exported from the WoSCC and Scopus databases and processed using the BibliometriX package in R, which is widely used for comprehensive science mapping and bibliometric analysis (29). The extracted information included publication year, authors, author affiliations, countries, journals, keywords, and citation data. Descriptive bibliometric metrics were calculated to examine the annual publication trends in this research field. BibliometriX was used to analyse keyword frequency and thematic evolution, allowing the identification of the most relevant keywords and the temporal evolution of research hotspots. Network visualization analyses were conducted using VOSviewer (version 1.6.20), a widely used tool for constructing and visualizing bibliometric networks (30). VOSviewer was used to construct collaborative networks among authors and among countries, enabling the identification of major collaboration patterns in this research field. In addition, a locally estimated scatterplot smoothing (LOESS) curve, together with its 95% confidence interval, was applied to visualize the temporal trend of annual publications.

## 3 Results

### 3.1 Global trends of scientific productivity

**Figure 2:**
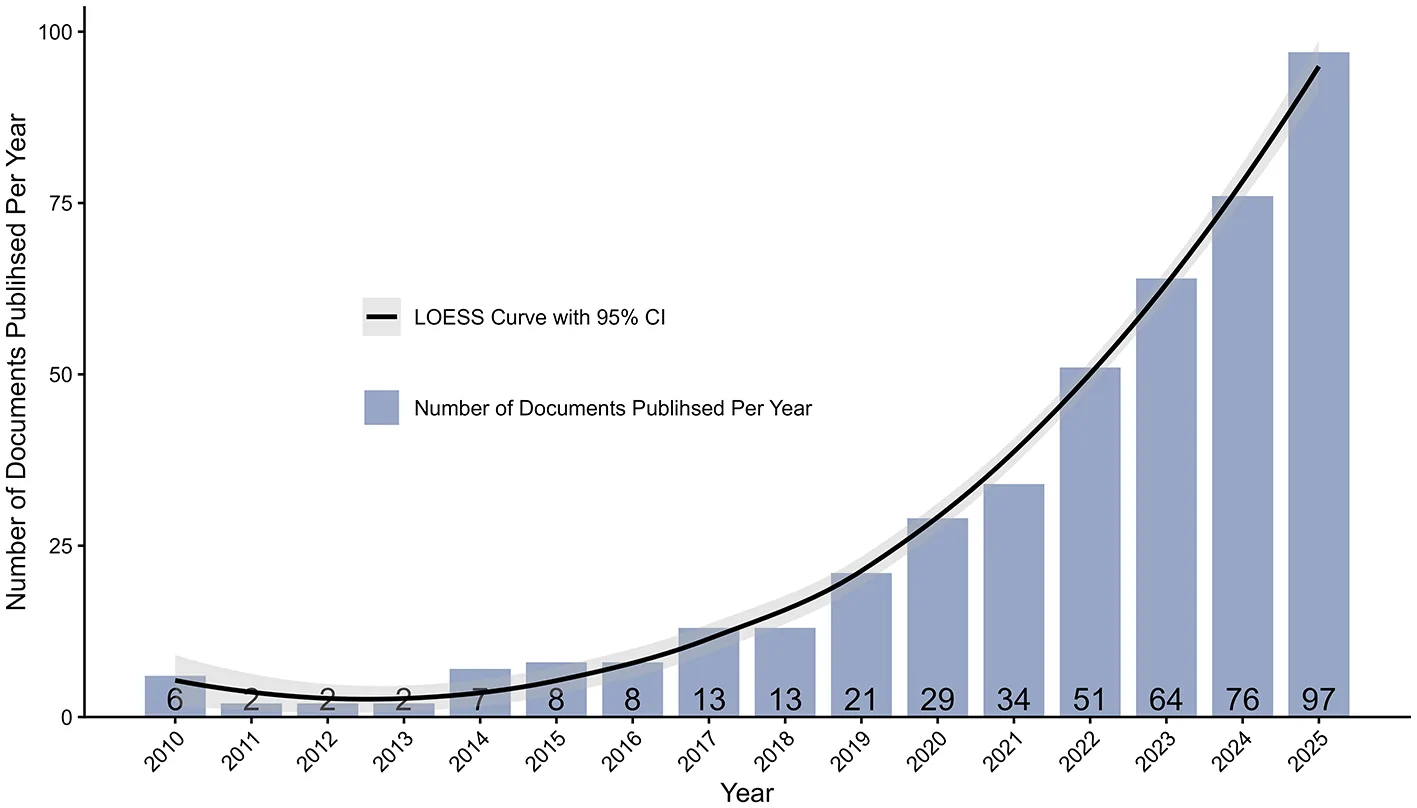
Annual number of publications in the field of virtual reality for musculoskeletal disorders from 2010 to 2025. The solid line represents the LOESS curve, and the shaded area represents the 95% confidence interval.

A total of 433 documents in the research field of VR for MSD were published between 2010 and 2025. These publications received 8,160 citations, with an average of 18.85 citations per publication. In terms of the publication type, these publications comprised 332 empirical studies and 101 reviews. Figure 2 illustrates the annual number of publications in this research field, along with a LOESS curve and its 95% confidence interval, which were used to visualize the overall growth trend. Overall, the number of publications shows a clear and sustained upward trajectory from 2010 to 2025. During the early stage of the research field (2010 to 2016), the annual number of publications remained relatively low, with fewer than ten publications per year. The annual number of publications first exceeded ten in 2017, indicating the nascent stage of research in this field. After 2018, the growth of annual number of publications accelerated rapidly. The annual number of publications increased steadily from 21 in 2019 to 51 in 2022, reflecting growing interest in this research field. This upward trend continued in subsequent years, with 64 publications in 2023, 76 in 2024, and 97 in 2025, representing the highest annual number of publications observed from 2010 to 2025. The LOESS curve indicates that annual number of publications increased gradually in the early years and accelerated in the later decade. This trend reflects the increasing interest in this research field and suggests that scientific productivity in this field is likely to continue expanding in the coming years.

### 3.2 Performance and the collaborative network of authors

**Figure 3:**
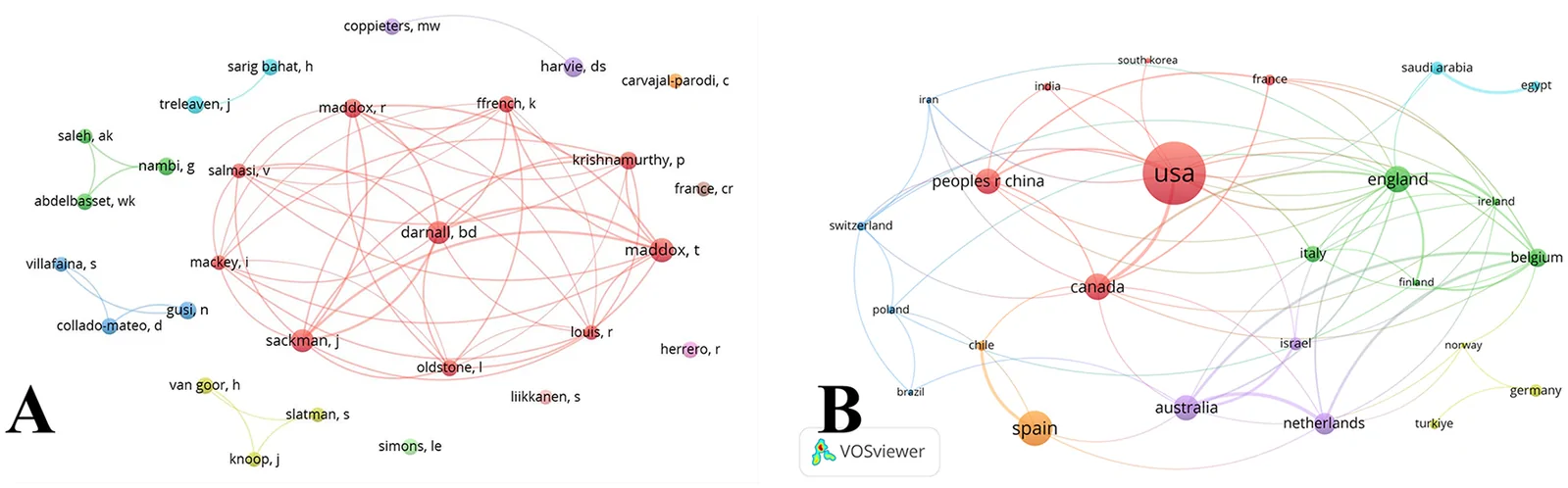
Collaboration networks in the field of virtual reality for musculoskeletal disorders from 2010 to 2025. **(A)** Author collaboration network. **(B)** Country collaboration network.

A total of 1,878 authors contributed to at least one publication in the field of VR for MSD. Table 1 presents the authors with the highest number of publications in this research field from 2010 to 2025. Maddox T. published the highest number of documents (13 items), followed by Sackman J and Darnall BD, each with 12 publications. In terms of citation impact, Krishnamurthy, P. had the highest average citation per item (ACI = 58.71), indicating a relatively strong influence of this author's publications. Other authors with relatively high citation impact included Sarig Bahat H (ACI = 52.17) and Treleaven J (ACI = 49.71). Figure 3A illustrates the collaborative network of authors in this research field. A total of 11 collaborative clusters were identified, indicating several independent research groups within this field. The largest cluster (red cluster) consisted of 10 authors, with Sackman J and Maddox T occupying central positions in this cluster. These authors showed strong collaborative links with several others in the cluster, suggesting that they play an important role in connecting different researchers within this research field. In addition to the largest cluster, several smaller collaborative groups were also observed. Overall, the collaborative network of authors suggests that research on VR for MSD is conducted by several collaborative groups, with a few key authors playing central roles in the development of this research field.

**Table 1:** Top 18 authors with the highest number of publications in the field of virtual reality for musculoskeletal disorders (2010–2025).

Rank	Author	Quantity	ACI	TLS
1	Maddox T	13	25.08	64
=2	Sackman J	12	26.75	64
=2	Darnall BD	12	35.25	61
4	Harvie DS	9	19.78	3
5	Maddox R	8	10.63	46
=6	Krishnamurthy P	7	58.71	40
=6	Gusi N	7	29.29	11
=6	Nambi G	7	24.29	11
=6	Treleaven J	7	49.71	5
=10	Ffrench K	6	10.83	37
=10	Oldstone L	6	10.83	37
=10	Abdelbasset WK	6	25.00	10
=10	Collado-mateo D	6	32.33	10
=10	van Goor H	6	21.00	7
=10	Sarig Bahat H	6	52.17	5
=10	Coppieters MW	6	8.50	3
=10	Herrero R	6	46.67	0
=10	Simons LE	6	17.17	0

ACI, Average Citation per Item; TLS, Total link strength with another authors.

### 3.3 Performance and the collaborative networks of countries

A total of 60 countries contributed to at least one publication in the field of VR for MSD. The top ten countries with the highest number of publications are presented in Table 2. The USA was the most productive country, with 94 publications, followed by Spain (46 publications). In terms of citation impact, Israel had the highest ACI (33.93), followed by Australia (ACI = 31.74) and Belgium (ACI = 28.29). Figure 3B presents the international collaborative network among countries. A total of seven collaboration clusters were identified, indicating several groups of countries that frequently collaborate with each other. The USA (red cluster) formed the largest collaborative group, including six countries, and showed strong connections with several other countries. England (green cluster) led another major collaboration cluster consisting of five countries. These results indicate that international collaboration in this research field is largely dominated by high-income countries, especially the USA and England. Although some middle-income countries, such as China and Saudi Arabia, also contributed, their participation in international collaborations appears to be relatively limited. Moreover, collaborations tend to occur within the same regions.

**Table 2:** Top eleven countries with the highest number of publications in the field of virtual reality for musculoskeletal disorders (2010–2025).

Rank	Country	Quantity	ACI	TLS
1	USA	94	26.69	30
2	Spain	46	18.37	12
=3	England	33	11.61	36
=3	Canada	33	14.24	27
=5	Australia	31	31.74	33
=5	China	31	16.06	14
7	Netherlands	26	11.31	25
8	Belgium	21	28.29	29
9	Italy	18	10.28	7
=10	Saudi Arabia	14	14.71	11
=10	Isreal	14	33.93	10

ACI, Average Citation per Item; TLS, Total link strength with another authors.

### 3.4 Performance of journals

A total of 433 documents in the field of VR for MSD were published across 207 different journals, indicating that this research field spans multiple academic disciplines. Table 3 presents the top ten journals with the highest number of publications. Among these journals, *Games for Health Journal* published the highest number of documents (15 items), followed by the *Journal of Medical Internet Research* (14 items). In terms of journal impact factor (IF), the *Journal of Medical Internet Research* had the highest IF in 2024 (IF = 6.0) among the listed journals, followed by *Virtual Reality* (IF = 5.0) and *JMIR Serious Games* (IF = 4.1). Most of the other journals had IF between 2 and 4, suggesting that research on VR for MSD is mainly published in specialized journals related to digital health, rehabilitation, and pain research.

**Table 3:** Top ten journals publishing the highest number of publications in the field of virtual reality for musculoskeletal disorders (2010–2025).

Rank	Journal	Quantity	2024 IF
1	Games for Health Journal	15	2.8
2	Journal of Medical Internet Research	14	6.0
=3	Frontiers in Virtual Reality	12	3.6
=3	JMIR Serious Games	12	4.1
=3	Journal of Clinical Medicine	12	2.9
6	Virtual Reality	11	5.0
7	Frontiers in Pain Research	8	2.7
=8	Applied Science - Basel	7	2.5
=8	The Clinical Journal of Pain	7	3.1
=8	Jmir Formative Research	7	2.1

2024 IF, 2024 Journal Impact Factor from Journal Citation Reports™.

### 3.5 Most cited publications

Table 4 lists the five most cited publications in the field of VR for MSD from 2010 to 2025. These highly cited papers mainly focus on the application of VR for pain management. The most cited publication was the systematic review and meta-analysis by Mallari et al. (31), titled “*Virtual reality as an analgesic for acute and chronic pain in adults: a systematic review and meta-analysis”*, which received 264 citations in total. This study provided comprehensive evidence on the effectiveness of VR in pain reduction. The second most cited paper was written by Pourmand et al. (32), which explored the use of VR as a clinical tool for pain management, with 216 total citations. Another highly cited study was conducted by Palazzo et al. (33), which examined barriers to adherence to home-based exercise VR programs for patients with chronic low back pain, highlighting the potential role of new technologies in improving rehabilitation adherence. Two additional influential review papers were published by Ahmadpour et al. (34) and Gupta et al. (35), both focusing on the mechanisms and clinical applications of VR in pain management. These studies discussed how VR may reduce pain through mechanisms such as distraction and cognitive engagement. Overall, the most cited publications indicate that pain management and the therapeutic mechanisms of VR are key research themes in this field.

**Table 4:** Top five publications with the highest number of citations in the field of virtual reality for musculoskeletal disorders (2010–2025).

Rank	Citations	Citation per year	First author	Year	Tittle	Resource	Type
1	264	33	Mallari B	2019	Virtual reality as an analgesic for acute and chronic pain in adults: a systematic review and meta-analysis	Journal of Pain Research	Review
2	216	24	Pourmand A	2018	Virtual Reality as a Clinical Tool for Pain Management	Current Pain and Headache Reports	Empirical study
3	205	18.64	Palazzo C	2016	Barriers to home-based exercise program adherence with chronic low back pain: Patient expectations regarding new technologies	Annals of Physical and Rehabilitation Medicine	Empirical study
4	190	23.75	Ahmadpour N	2019	Virtual Reality interventions for acute and chronic pain management	The International Journal of Biochemistry & Cell Biology	Review
5	188	20.89	Gupta A	2018	Innovative Technology Using Virtual Reality in the Treatment of Pain: Does It Reduce Pain via Distraction, or Is There More to It?	Pain Medicine	Review

### 3.6 Performance of keywords

**Figure 4:**
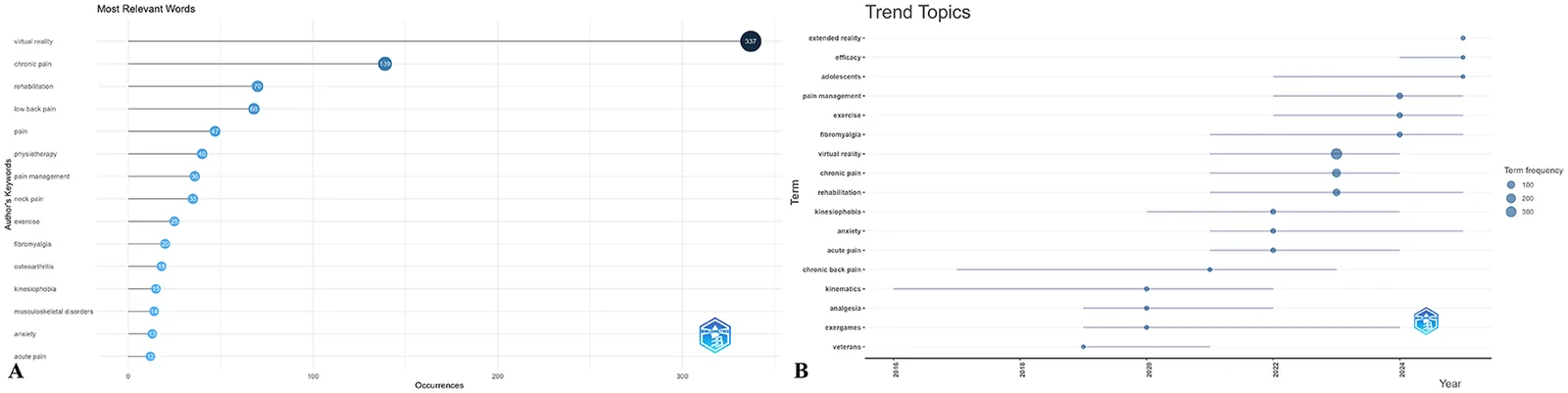
Keyword analysis in the field of virtual reality for musculoskeletal disorders. **(A)** Top 15 most frequently used keywords. **(B)** Evolution of keywords over time.

Figure 4A presents the most frequently used author keywords, excluding the general term “virtual reality.” Among the remaining keywords, “chronic pain” (139 occurrences) was the most frequently used keywords, indicating that pain management is a hotspot in this research field. Other frequently reported keywords included “rehabilitation” (70 occurrences) and “low back pain” (68 occurrences), suggesting that many studies have explored rehabilitation interventions for patients with musculoskeletal pain conditions. Additional commonly used keywords included “pain” (47 occurrences), “physiotherapy” (40 occurrences), and “pain management” (36 occurrences). Several keywords related to specific musculoskeletal conditions were also identified, such as “neck pain,” “fibromyalgia,” and “osteoarthritis,” indicating that research has examined a variety of MSDs. Overall, these keywords suggest that this research field mainly focuses on pain-related conditions and rehabilitation interventions for MSD from 2010 to 2025.

To explore the emerging research hotspots, Figure 4B illustrates the evolution of keywords over time. Earlier studies (around 2016 to 2019) mainly addressed topics such as kinematics, analgesia, and exergames, which were often used to explore movement-related outcomes and pain distraction. After 2020, the focus gradually shifted toward clinical rehabilitation and pain-related conditions, with more studies examining chronic pain, fibromyalgia, exercise, and rehabilitation. In recent years (2022 to 2024), keywords such as pain management, adolescents, efficacy, and extended reality have appeared more frequently. Overall, these trends indicate a shift from early technological exploration toward the clinical application and effectiveness of technology-assisted interventions for MSD.

## 4 Discussion

This bibliometric analysis provides an overview of the development of research on VR for MSD from 2010 to 2025. The results show a steady increase in the annual number of publications over the study period, with particularly rapid growth observed after 2019. This trend suggests that research on VR for MSD has gradually evolved from an emerging research topic into a more active area of investigation (36), likely driven by advances in VR technology and increasing clinical interest in innovative approaches for rehabilitation and pain management (37).

### 4.1 Global productivity of research

The annual number of publications remained relatively low during the early development of research on VR for MSD (2010 to 2016), with fewer than ten articles published each year. This pattern is typical for emerging research fields, where scientific interest is still developing and methodological approaches are being explored (38). From 2017, the number of publications in this research field began to increase steadily, with a more rapid acceleration after 2019. By 2025, the annual number of publications reached 97 publications, representing the highest level observed during the study period. The LOESS curve also indicates a clear increasing trajectory in recent years, suggesting that research in this research field has entered a period of rapid development. A similar growth pattern has also been observed in other digital health fields, such as mobile health and digital therapeutics (39), and VR for mental health (38). Several factors may explain this growth. One important factor is the rapid advancement and increased accessibility of VR technologies. Early clinical VR systems were expensive and technically complex, which limited their use to laboratory environments only. However, the development of affordable head-mounted displays and portable VR platforms has made immersive technologies more accessible for both clinical and home-based rehabilitation settings. Rizzo and Koenig (40) proposed that improvements in hardware affordability and usability have played a critical role in enabling the translation of VR from laboratory settings into clinical practice. As a result, VR interventions can now be implemented more easily in rehabilitation programs and remote therapeutic environments. Another factor contributing to the expansion of research is the growing clinical demand for innovative approaches to manage MSD. MSDs are among the leading causes of disability worldwide and represent a major burden for healthcare systems (41). The majority of patients experience persistent pain, reduced physical function, and poor adherence to long-term rehabilitation programs (42). Therefore, VR-based interventions have attracted attention because they can provide immersive and interactive environments that enhance patient engagement during therapy (43). Previous studies have shown that VR can reduce pain intensity and improve adherence to exercises through mechanisms such as distraction, cognitive engagement, and increased motivation (31). In sum, the steady increase in publications observed in this study suggests that VR for MSD is evolving from an exploratory research topic into a more established area within digital rehabilitation research. The recent increase in publications also indicates that VR-based interventions are gaining increasing recognition as a promising component of rehabilitation and management of MSD.

### 4.2 Performance of authors, countries, and journals

The authorship analysis shows that research in the field of VR for MSD is unevenly distributed, with a relatively small group of researchers contributing a large proportion of publications. Such patterns are commonly observed in emerging research fields, where early scientific development is often driven by a limited number of leading research groups that establish the initial methodological and clinical frameworks (44). The collaborative structure observed in the co-authorship network may also reflect the interdisciplinary nature of research in this field. Studies in this field often require collaboration between clinicians, rehabilitation scientists, psychologists, and engineers, as well as access to specialized equipment and software development resources (40, 45). As a result, research activities tend to be organized around established interdisciplinary teams rather than isolated individual researchers. Similar collaboration patterns have been reported in other technology-driven medical research fields, where multidisciplinary expertise is required to translate technological innovation into clinical practice (46).

At the country level, research productivity is largely concentrated in high-income countries, particularly the USA and several European nations. The USA produced the largest number of publications, while countries such as Australia and Belgium demonstrated relatively high citation impact. This distribution is consistent with previous bibliometric studies of digital health and rehabilitation technologies, which have shown that research output is often strongly associated with national research capacity, technological infrastructure, and funding availability (24). Countries with well-established digital health ecosystems and strong academic research institutions are therefore more likely to lead the development and evaluation of VR-based interventions. Despite this concentration of research activity in high-income settings, MSDs represent a major health challenge worldwide. According to the Global Burden of Disease study, MSDs are among the leading causes of disability globally and account for a substantial proportion of years lived with disability across all regions (41). Importantly, a large share of this burden occurs in low- and middle-income countries, where access to rehabilitation services and specialized care remains limited (47). This mismatch between disease burden and research production suggests that many regions most affected by MSD may have limited participation in the development of emerging rehabilitation technologies. International collaboration patterns also appear to follow regional structures. The network analysis shows that collaboration often occurs between countries within the same geographical regions rather than across distant regions. Similar regional collaboration patterns have been observed in other bibliometric analyses of digital health research (48). Strengthening cross-regional collaborations may therefore help promote knowledge exchange and improve the global accessibility of VR-based rehabilitation interventions.

The journal distribution further highlights the interdisciplinary character of this research field. The 433 publications were distributed across 207 different journals, indicating that research on VR for MSD spans multiple scientific domains. Many of the most active journals, including *Games for Health Journal* and the *Journal of Medical Internet Research*, focus on digital health technologies and interactive medical applications. Other journals, such as *JMIR Serious Games* and *Virtual Reality*, reflect the technological dimension of the field, while journals like *Journal of Clinical Medicine* represent the clinical and rehabilitation perspectives. This broad journal distribution suggests that VR for MSD research sits at the intersection of digital health, rehabilitation science, and pain research.

Overall, these findings indicate that VR for MSD research is shaped by interdisciplinary collaboration, technological innovation, and global disparities in research capacity. Continued international collaboration and broader participation from low- and middle-income countries may help accelerate the development and implementation of VR-based rehabilitation strategies worldwide.

### 4.3 Research trends and hotspots

Keyword analysis helps explain what this field has mainly been studying. After excluding the general term “virtual reality,” the most frequent keywords were chronic pain, rehabilitation, and low back pain, which suggests that the literature has been centered on pain management and functional recovery rather than on the technology itself. This pattern is consistent with previous reviews showing that VR has mainly been investigated as a non-pharmacological tool for pain relief and rehabilitation in MSDs, especially chronic pain, back pain, and related functional limitations (16, 17, 27, 31).

The earlier appearance of terms such as kinematics, analgesia, and exergames reflects a stage in which researchers were still examining the basic therapeutic value of immersive technologies. Early studies mainly focused on whether VR could reduce pain through attentional distraction and multisensory stimulation, and whether interactive game-based environments could support movement training and increase engagement during rehabilitation exercises (45, 49). This early focus aligns with the way VR has been described in recent literature, namely as a tool that can redirect attention, deliver multisensory stimulation, and create controlled interactive environments that influence both perception and motor behavior. Emerging evidence indicates that immersive VR may modulate pain perception through attentional distraction and multisensory integration, while simultaneously supporting motor learning and behavioral engagement during rehabilitation (50).

After 2020, the keywords became more clinically oriented. Terms such as chronic pain, exercise, and rehabilitation appeared more frequently, suggesting that research was gradually shifting from proof-of-concept studies toward the evaluation of treatment effects. This transition is consistent with the broader development of VR interventions in rehabilitation research. Recent systematic reviews and clinical studies increasingly assess VR in terms of pain reduction, physical function, balance performance, disability, and psychological outcomes across different musculoskeletal conditions, rather than focusing primarily on the technological aspects of the intervention (31, 51). This shift may reflect an improved understanding of the therapeutic mechanisms underlying VR interventions. Beyond providing an engaging rehabilitation environment, immersive VR may reduce pain perception by redirecting attention away from nociceptive input and by altering the integration of visual, somatosensory, and proprioceptive information. In parallel, repeated task-oriented practice and real-time visual feedback may facilitate motor learning and movement control, while the immersive and interactive nature of VR may enhance motivation, behavioral engagement, and adherence to rehabilitation programmes, thereby contributing to improved functional outcomes (49, 52, 53).

Recent keywords such as pain management, efficacy, and extended reality suggest that the field is moving beyond the question of whether VR is engaging and toward the question of how VR may exert its therapeutic effects in clinical practice. Accordingly, increasing attention has been directed toward the neurophysiological mechanisms underlying VR-based rehabilitation. Chronic pain has been associated with functional and structural alterations in cortical and subcortical regions involved in sensory-discriminative, cognitive–affective, and sensorimotor aspects of pain, including the prefrontal cortex, anterior cingulate cortex, insula, somatosensory cortices, and motor-related regions (54, 55). By creating immersive environments that manipulate attention, embodiment, and visual–proprioceptive feedback, VR may influence the processing of pain-related sensory information and modify maladaptive body representation and the processing of movement-related pain. Such effects provide a plausible mechanism through which VR could affect both the cognitive–affective and sensorimotor dimensions of chronic pain (55). Furthermore, congruent multisensory and visuomotor feedback may facilitate sensorimotor recalibration and adaptive changes in body representation, which may in turn support improvements in movement confidence, movement quality, and functional recovery (56). Moreover, in patients with MSD, VR-based therapy has been associated not only with reductions in pain intensity and disability, but also with reported alterations in brain networks related to pain processing and motor control, including changes in prefrontal and somatomotor connectivity as well as white-matter microstructure (57). In addition, experimental studies have shown that manipulating visual–proprioceptive feedback in immersive environments can alter movement-evoked pain responses in individuals with chronic low back pain, suggesting that VR may influence body representation and the processing of movement-related pain (58). Nevertheless, direct evidence that these clinical improvements are mediated by specific cortical changes remains limited in the field of VR for musculoskeletal disorders, and further studies combining clinical outcomes with neuroimaging and neurophysiological measures are required. Taken together, these findings suggest that VR should be regarded not merely as a distraction technique but as a multimodal intervention with the potential to promote neuroplastic adaptations through the integration of cognitive, sensorimotor, and affective processes, thereby supporting sustained improvements in pain, motor control, and physical function.

In summary, the keyword patterns suggest three main research phases: an early stage focusing on technological feasibility and pain distraction, a transitional stage emphasizing rehabilitation and chronic pain management, and a recent stage centered on clinical effectiveness, mechanisms of action, and the application of extended reality technologies. These findings indicate that the field is gradually shifting from technology-driven exploration to clinically oriented and mechanism-informed research. Future studies are likely to place greater emphasis on elucidating the neurophysiological mechanisms underlying VR interventions by integrating neuroimaging and neurophysiological techniques with clinical outcome measures. Such approaches may facilitate the development of more targeted, mechanism-informed, and evidence-based VR rehabilitation strategies for musculoskeletal disorders.

### 4.4 Limitation and future studies

This study has several limitations that should be considered when interpreting the findings. First, although two large multidisciplinary databases (WoSCC and Scopus) were used to improve literature coverage, relevant publications indexed only in other databases, such as PubMed or Embase, may have been missed. Differences in database coverage and indexing practices have been widely documented and may influence bibliometric results (24). Second, only English-language publications were included, which may lead to the underrepresentation of studies conducted in non-English-speaking regions. Third, bibliometric analyses rely largely on citation-based indicators. Citation counts reflect the visibility and influence of publications rather than the methodological quality or clinical effectiveness of individual studies. Highly cited papers may represent early or influential contributions rather than the strongest clinical evidence (59). Fourth, database indexing is subject to time delays. As a result, records from the most recent year, particularly 2025, may not fully capture all publications in this field and could lead to a slight underestimation of recent research activity (60). Finally, although tools such as BibliometriX and VOSviewer allow the visualization of collaboration networks and keyword trends, the interpretation of clusters and thematic structures inevitably involves some degree of subjective judgement. Future research could therefore combine bibliometric analysis with systematic reviews or meta-analyses to better assess the clinical effectiveness and implementation of VR-based interventions for MSD.

## 5 Conclusion

This bibliometric analysis provides a comprehensive overview of the development of VR for MSD from 2010 to 2025. The findings demonstrate a steady increase in scientific productivity, particularly after 2019, reflecting growing interest in VR-based rehabilitation and pain management. Research in this field is characterized by interdisciplinary collaboration and is predominantly led by high-income countries. Keyword analysis further indicates that the field has evolved from early technology-driven exploration toward clinically oriented and mechanism-informed research, with increasing attention directed to pain management, rehabilitation outcomes, and the neurophysiological mechanisms underlying VR interventions. These findings not only map the knowledge structure and emerging research trends in this field but also provide a foundation for future interdisciplinary research and the development of more targeted and evidence-based VR rehabilitation strategies for MSD.

## Data Availability

The original contributions presented in the study are included in the article/Supplementary material, further inquiries can be directed to the corresponding author.
